# Involvement of inflammatory responses in the brain to the onset of major depressive disorder due to stress exposure

**DOI:** 10.3389/fnagi.2022.934346

**Published:** 2022-07-22

**Authors:** Shingo Miyata, Yugo Ishino, Shoko Shimizu, Masaya Tohyama

**Affiliations:** ^1^Division of Molecular Brain Science, Research Institute of Traditional Asian Medicine, Kindai University, Osaka, Japan; ^2^Osaka Prefectural Hospital Organization, Osaka, Japan

**Keywords:** major depressive disorders, stress hypothesis, cGAS-STING pathway, inflammasome, immunosenescence

## Abstract

Major depressive disorder (MDD) is a multifactorial disease affected by several environmental factors. Although several potential onset hypotheses have been identified, the molecular mechanisms underlying the pathogenesis of this disorder remain unclear. Several recent studies have suggested that among many environmental factors, inflammation and immune abnormalities in the brain or the peripheral tissues are associated with the onset of MDDs. Furthermore, several stress-related hypotheses have been proposed to explain the onset of MDDs. Thus, inflammation or immune abnormalities can be considered stress responses that occur within the brain or other tissues and are regarded as one of the mechanisms underlying the stress hypothesis of MDDs. Therefore, we introduce several current advances in inflammation studies in the brain that might be related to the pathophysiology of MDD due to stress exposure in this review.

## Introduction

One of the major mood disorders, major depressive disorder (MDD), is known to show significant morbidity and is associated with social and economic disability [Smith, 2014; Vigo et al., 2016; World Health Organization (WHO), 2020; Wang et al., 2022]. MDD is believed to be a multifactorial disease attributable to both environmental and genetic factors, although the genes responsible for and the molecular mechanisms underlying the pathogenesis of MDD remain unclear (Lopizzo et al., 2015; Tohyama et al., 2015; Miyata et al., 2015a). Although selective serotonin reuptake inhibitors (SSRIs) and serotonin–norepinephrine reuptake inhibitors (SNRIs), which are therapeutic agents for MDD, are based on the most widely accepted hypothesis, the monoamine hypothesis, they do not exhibit adequate therapeutic effects in approximately one-third to half of patients with MDD (Hieronymus et al., 2016; Jesulola et al., 2018; Zhao et al., 2022). The important findings of low-dose ketamine effects have been recently suggested to provide more insights into this discrepancy. Administration of low-dose ketamine increases spine numbers on the dendrite in a short time and shows a sustained antidepressant effect within 1 h not only neurons but also astrocytes (Pryazhnikov et al., 2018; Zanos and Gould, 2018; Stenovec et al., 2021). Thus, this change in the neuronal plasticity and glia functions in the brain might underlie the pathophysiology of MDD and may be related to the monoamine hypothesis.

Considering the relationship between the neuronal plasticity (or synaptic function) and the pathology of MDD, a thorough understanding of the response to inflammation in the brain is important. Several recent studies have suggested that among many environmental factors, inflammation and immune abnormalities in the brain and peripheral tissues are associated with the onset of neuropsychiatric disorders, and inflammation during brain development has been shown to strongly increase the risk of MDD [Goldsmith et al., 2016; Pape et al., 2019; Abdoli et al., 2020; World Health Organization (WHO), 2020; Hansen, 2022; Wang et al., 2022]. In addition, synaptic pruning is induced by increased reactive microglia in the brain of patients with MDD, and nonsteroidal anti-inflammatory drugs (NSAIDs) perform assistant functions in enhancing the therapeutic effects in patients with senile depression (Wohleb et al., 2016; Yuan et al., 2020; Hang et al., 2021; Dawood et al., 2022; Li et al., 2022; Strekalova et al., 2022). Changes in the expression levels of cytokines and chemokines have also been reported to occur in the blood of patients with MDD (Martinuzzi et al., 2021; Poletti et al., 2021; Agarwal et al., 2022).

Among the multiple environmental factors, repeated stressful events are associated with the onset of MDD, and stress has been shown to activate the hypothalamic–pituitary–adrenocortical (HPA) system (Pariante and Lightman, 2008; Nouraei et al., 2018; Ceruso et al., 2020; Hennings et al., 2021). The negative feedback of corticosteroids on the HPA system occurs at the level of the hypothalamus and anterior pituitary *via* glucocorticoid receptors (GRs). Dysregulation of this negative feedback mechanism has been reported in patients with MDD, which results in hyperactivity of the HPA system and higher basal levels of serum corticosterone (Almeida et al., 2021). In addition, many clinical studies have demonstrated that elevated corticosterone levels trigger depressive symptoms (Short et al., 2016; Raupp-Barcaro et al., 2018). These facts strongly indicate that sustained elevated levels of plasma corticosteroids are a cause of MDD. However, glucocorticoids (humans, cortisol, rodents, and corticosterone) can show both inflammation-aggravating and anti-inflammatory effects. One of the inflammatory cytokines, interleukin-1 (IL-1), inhibits the translocation of the GR from the cytoplasm to the nucleus (Wang et al., 2004). Furthermore, in the activation of the sympathetic nervous system, which is another stress response system, noradrenaline has been reported to act on the microglia to induce IL-1β mRNA expression (Sugama et al., 2019; Tozaki-Saitoh et al., 2020).

As a part of the molecular mechanism of the onset of depression based on this stress hypothesis, microglial activation, and intracerebral inflammation have been shown to be important for the emotional changes caused by chronic stress in rodent models (Furuyashiki et al., 2019). Chronic stress stimulates IL-1β production, and cyclooxygenase (COX) 1 is involved in the biosynthesis of the inflammation-related molecule prostaglandin (PG) E2 following chronic stress exposure (Nie et al., 2018). IL-1β and COX-1 are mainly expressed in microglia in the brain; however, the relationship between the changes in oligodendrocyte and myelin functions and the response to inflammation in the brain is largely unknown. Therefore, this mini-review focuses on the current advances in inflammation studies in the brain that might be related to the pathophysiology of MDD due to stress exposure, which seems to be related to not only neuronal functions but also oligodendrocytes and myelin functions.

### Promote or suppress: Glucocorticoid and noradrenaline

Activated microglia have been reported to be involved in various pathologies, including depression, bipolar disorder, and sleep disorder (Stokes et al., 2015; Tay et al., 2018). Stress exposure has been shown to be associated with microglial activation (Walker et al., 2013; Sugama and Kakinuma, 2020). Stress-induced microglial activation involves not only acute stress exposure but also chronic stress exposure. Furthermore, activation of both the HPA axis and the sympathetic nervous system occurs with stress exposure (Elsaafien et al., 2021; Sjörs Dahlman et al., 2021). GR and α- and β-adrenergic receptors (ARs) are expressed in microglia and are involved in the morphological and functional changes of microglia with stress exposure; however, glucocorticoid and noradrenaline functions for microglial activity and inflammation after stress exposure are mediated by contradictory mechanisms (Wohleb et al., 2011; Walker et al., 2013; Delpech et al., 2015; Barnard et al., 2019; Ryan et al., 2020; Brás et al., 2022).

#### Glucocorticoids

Our previous studies have indicated that elevation of glucocorticoid levels by chronic stress exposure induces the expression of adhesion molecules in oligodendrocytes *via* the activation of the PI3K–PDK1–SGK1–NDRG1 pathway, which causes excess arborization of oligodendrocytes (Miyata et al., 2011). However, no reduction in the number of oligodendrocytes was observed in the corpus callosum. We also found that the nodes and paranodes of Ranvier in the corpus callosum were narrower and oligodendrocyte activity decreased after chronic stress exposure (Miyata et al., 2015b, 2016). However, we could not find microglial activation in the corpus callosum for 24 h after the last stress exposure (Miyata et al., 2011). A recent report suggested that microglia are transiently activated for 1.5–4 h after social defeat stress, and this microglial activation is enhanced in the hippocampus and medial prefrontal cortex (PFC) of mice exposed to chronic stress (Nie et al., 2018; Furuyashiki and Kitaoka, 2019). Thus, this stress response to microglia is transient and shows specificity for different brain regions; therefore, further investigation of the relationship between the elevation of plasma glucocorticoid levels, the inflammatory response in the brain and peripheral tissues, and the depression-like pathology is required.

The contradictory mechanisms of glucocorticoids function in both microglial activity and inflammation after stress exposure. Previous reports have presented several hypotheses regarding this controversial phenomenon. The priming hypothesis indicates that the inflammation state is exacerbated by glucocorticoid treatment of microglia before lipopolysaccharide (LPS) stimulation; however, microglial activation is inhibited by glucocorticoid treatment of microglia after LPS stimulation (Frank et al., 2010; Carrillo-de Sauvage et al., 2013). The next hypothesis postulates a dependency on glucocorticoid concentration. Treatment of microglia with a low concentration of glucocorticoids indicates suppression of inflammation; however, treatment with a high concentration of glucocorticoids for microglia promotes inflammation (MacPherson et al., 2005; Liu et al., 2018b).

#### Noradrenaline

The contradictory mechanisms underlying noradrenaline also function in both microglial activity and inflammation after stress exposure. The noradrenaline signal was shown to induce p38 MAPK- and ERK1/2-dependent inflammatory responses in the absence of LPS stimulation; however, after LPS stimulation, noradrenaline stimulates cAMP and suppresses the inflammatory response in a PKA-dependent manner (Qian et al., 2009; Takahashi et al., 2018; Zhang et al., 2018; Ryan et al., 2020).

### cGAS-STING pathway

The cyclic GMP–AMP synthase (cGAS)–stimulator of interferon genes (STING) pathway is one of the mechanisms by which DNA appears in the cytoplasm, induces innate immune systems, and strongly induces type I interferon (IFN-I) (Margolis et al., 2017; Sprooten et al., 2019; Taguchi and Mukai, 2019; Yang et al., 2022). In recent years, the response to autologous DNA leakage into the cytoplasm and the abnormal activation of the cGAS-STING pathway have been reported to be involved in many neurodegenerative and inflammatory diseases (Jauhari et al., 2020; Pal et al., 2020; Barrett et al., 2021; Jassim et al., 2021; Hinkle et al., 2022).

#### Type I interferon

The IFN therapy has been established as a useful treatment for many diseases such as hepatitis C, hepatitis B, and multiple myeloma (Ettari et al., 2016; Ye and Chen, 2021; Cao et al., 2022; Liu, H. et al., 2022). However, IFN-I treatment is known to induce various neuropsychiatric symptoms, and depression is known to occur at a high frequency of 30% or more (Tovey and Lallemand, 2010; Pinto and Andrade, 2016; Liu et al., 2020). Nevertheless, the core symptoms of IFN-induced depression are reported to be physical symptoms, such as sleep disorders and loss of appetite, and the mood symptoms that are observed in general depression are difficult to identify in the foreground, with few reports describing the molecular mechanisms underlying these differences. Thus, although the relationship between the cGAS-STING pathway and the onset of depression has not yet been reported, the primary goal of future research is the involvement of chronic stress in the endoplasmic reticulum and the Golgi apparatus functions as the molecular mechanism of INF-I production by TBK1-IRF3 pathway activation and the pathology of INF-I-specific depressive symptoms.

#### Senescence-associated secretory phenotype

Aging is well-known to cause changes leading to immune system decline, a process called “immunosenescence” (Pietrobon et al., 2020). In addition, a previous report has shown that the elderly have mild chronic inflammation and that inflammatory proteins and growth factors are constantly increasing in the elderly brain and a senescence-associated secretory phenotype (SASP) mediates the transport of these factors extracellularly (Gamage et al., 2020; Liao et al., 2021; Blagosklonny, 2022). However, the increased levels of inflammatory cytokines, such as TNF-α, IL-1, and IL-6, are very low in the elderly, and this mild chronic inflammation in the elderly, called “inflammaging,” is one of the health indicators of the elderly (Rea et al., 2018; Pietrobon et al., 2020). Thus, these phenomena in the elderly might be related to the activation of the SASP by transcription factors, such as NF-κB and C/EBP-β, and the onset of senile depression (Bruxel et al., 2019; Saito et al., 2021).

### Inflammasome

Inflammasomes are protein complexes that play a central role in innate immunity (Man and Kanneganti, 2016; Gasteiger et al., 2017; Challagundla et al., 2022; Dong et al., 2022). Inflammasome signals have been reported to cause the activation of caspase-1 and increase the secretion of TNF-α and IL-1β, which are inflammatory cytokines in microglia and may be associated with depressive symptoms (Zhu et al., 2017; Slusarczyk et al., 2018; Li et al., 2021; Rao et al., 2021; Liu Q. et al., 2022).

#### Nucleotide-binding, leucine-rich repeat, pyrin domain containing 3

Nucleotide-binding, leucine-rich repeat, pyrin domain containing 3 (NLRP3) has been reported to function as a “hub” for the pathophysiology of various neuropsychiatric disorders, including MDD (Iwata et al., 2016; Yamanashi et al., 2017; Zhang et al., 2022). A previous report has indicated that an increase in the ATP concentration in the PFC and hippocampus was observed after restraint stress or chronic mild stress as a model for the onset of depression and that ATP binds to the P2X7 receptor in microglia (Ribeiro et al., 2019; Lan et al., 2020; Drinkall et al., 2022). Next, this P2X7 signal decreases the concentration of intracellular potassium, inducing the formation of a protein complex between the NLRP3 oligomer, apoptosis-associated speck-like protein containing a CARD (ASC), and pro-caspase 1 (Yang et al., 2018; von Muecke-Heim et al., 2021). The suppression of inflammation in this inflammasome pathway might be a function of the antidepressant effect; however, many P2X7 receptor inhibitors have low central transferability, and IL-1β-neutralizing antibodies are extremely expensive (Iwata et al., 2016; Wang et al., 2016; Yue et al., 2017; Ren et al., 2021).

### Brain-related inflammation

Brain inflammation after chronic stress exposure involves not only the induction of inflammatory cytokines from microglia but also multiple peripheral reactions, such as dysfunction of the blood–brain barrier, involvement of IL-1 receptors in vascular endothelial cells, and promotion of monocyte migration from the peripheral tissues into the brain (Chaves-Filho et al., 2019; Kitaoka, 2022).

#### NSAIDs

Nonsteroidal anti-inflammatory drugs inhibit COX-1 and COX-2 functions and decrease prostaglandin and thromboxane levels, thus inducing a reduction in TNF-α and IL-6 levels (Wohleb et al., 2016; Yuan et al., 2020; Hang et al., 2021; Li et al., 2022). COX-1 selective inhibitors that suppress inflammation in the brain can inhibit depressive symptoms after chronic stress exposure (Guevara et al., 2015; Brkic et al., 2020). COX-1 is expressed selectively in the microglia, and it has been suggested to be involved in inflammation control and depression by microglial regulation. Toll-like receptors (TLRs) are highly expressed in microglia. Chronic stress exposure induces the TLR2/4-dependent inflammatory cytokines IL-1α and TNF-α in the microglia of PFC (Nie et al., 2018). Furthermore, TLR2/4 is not only required for enhanced expression of inflammatory cytokines in PFCs but also essential for PGE2 production in the subcutaneous region (Nie et al., 2019). The COX-2 selective inhibitor celecoxib inhibits prostaglandin and downstream cytokine production (Strekalova et al., 2022). Thus, several clinical studies have shown that celecoxib enhances the antidepressant effect; however, some studies have indicated that celecoxib might increase brain inflammation (Baune, 2017; Lang and Walter, 2017; Alboni et al., 2018; Strumila et al., 2022). These conflicting results may be resolved through more detailed basic and clinical studies in the future (Luning Prak et al., 2022).

#### IL-1β and IL-6

Corticosterone, which is closely related to chronic stress responses, increases the number of neutrophils in the bone marrow (Sarjan and Yajurvedi, 2018). Catecholamines, which are chronic stress response factors, promote the proliferation and recruitment of hematopoietic stem cells in the bone marrow (Hanns et al., 2019). Thus, blood neutrophil and monocyte counts increase in mice exposed to chronic stress.

Increased expression of CCL2, a CCR2 ligand and one of the chemokines involved in the mobilization of monocytes in the blood, was observed in microglia after chronic stress exposure, and increased expression of IL-1β was observed in monocytes in the brain (Slusarczyk et al., 2015; Zhao et al., 2016; McKim et al., 2018; Trojan et al., 2019). This increase in monocyte-derived Il-1β expression and activation by caspase-1 is associated with anxiety symptoms (Wohleb et al., 2015; McKim et al., 2018).

The relationship between IL-6 and MDD pathology has been reported previously (Maes et al., 2016; Ting et al., 2020; Kuo et al., 2021). Serum IL-6 concentration was significantly higher in patients with MDD and decreased only in the SSRI and SNRI reaction groups (Fei et al., 2021). Thus, high IL-6 levels in the blood are associated with intractable depression, and IL-6 levels in the blood may predict the therapeutic response to SSRIs and/or SNRIs.

### COVID-19 infections

Coronaviruses can damage the central nervous system (CNS) *via* direct invasion. These viruses enter *via* the blood-circulation pathway, neuronal pathway, and binding to angiotensin-converting enzyme-2 (ACE-2) receptors (Wu et al., 2020; Kumar et al., 2021; Hornick et al., 2022; Thye et al., 2022). Furthermore, some patients with COVID-19 show cytokine dysregulation, such as TNF-α, IL-1β, IL-6, and IFN-γ, which are well known to be associated with MDD (Liu et al., 2021; Lorkiewicz and Waszkiewicz, 2021). Cytokine-release syndrome or COVID-19 infection-induced inflammatory responses may be associated with chronic stress-related inflammatory responses that prolong and increase post-viral symptoms (Troyer et al., 2020). Furthermore, some COVID-19 survivors show a relationship between disruption of the HPA axis and several psychiatric symptoms, including anxiety, depression, and post-traumatic stress disorder (PTSD) (Hu et al., 2020; Badenoch et al., 2021; Mohammadkhanizadeh and Nikbakht, 2021; Hornick et al., 2022; Thye et al., 2022).

## Conclusion

Many diseases of the nervous system attack nonneuronal cells and indirectly affect neuronal function and integrity. We have already demonstrated for the first time both *in vivo* and *in vitro* that exposure to chronic stress leads to alterations in oligodendrocyte activity and disrupts axon-myelin adhesion at the paranodes and nodes of Ranvier, as evidenced by the diffuse distribution of key proteins and reduced axonal activity (Miyata et al., 2011, 2015b, 2016). We further found changes in some factors involved in the inflammatory response based on the results of our recent study. Therefore, we have continued investigating the relationship between brain inflammation and depressive pathology.

Compromised white matter/myelin integrity has been reported not only in patients with MDD but also in animal models of MDD (Miyata et al., 2016; Miyata, 2019; Greenberg et al., 2021). Furthermore, in brain imaging and postmortem evaluations of the human brain, patients with MDD have been found to show abnormalities in the white matter and/or oligodendrocytes (Czéh and Nagy, 2018; Vostrikov and Uranova, 2020). Interestingly, stress exposure in animal models can decrease the number of oligodendrocytes in the cortex and amygdala, indicating potential links between disturbed myelination and MDD (Liu et al., 2018a; Kokkosis et al., 2022; Madeira et al., 2022). In contrast, it has been reported that astrocytes are related to neuroinflammation, and astrocyte density in the frontal region is reduced in chronic stress conditions (Banasr et al., 2010; Hattori et al., 2017; Takarada-Iemata et al., 2018; Roboon et al., 2021).

**Figure 1:**
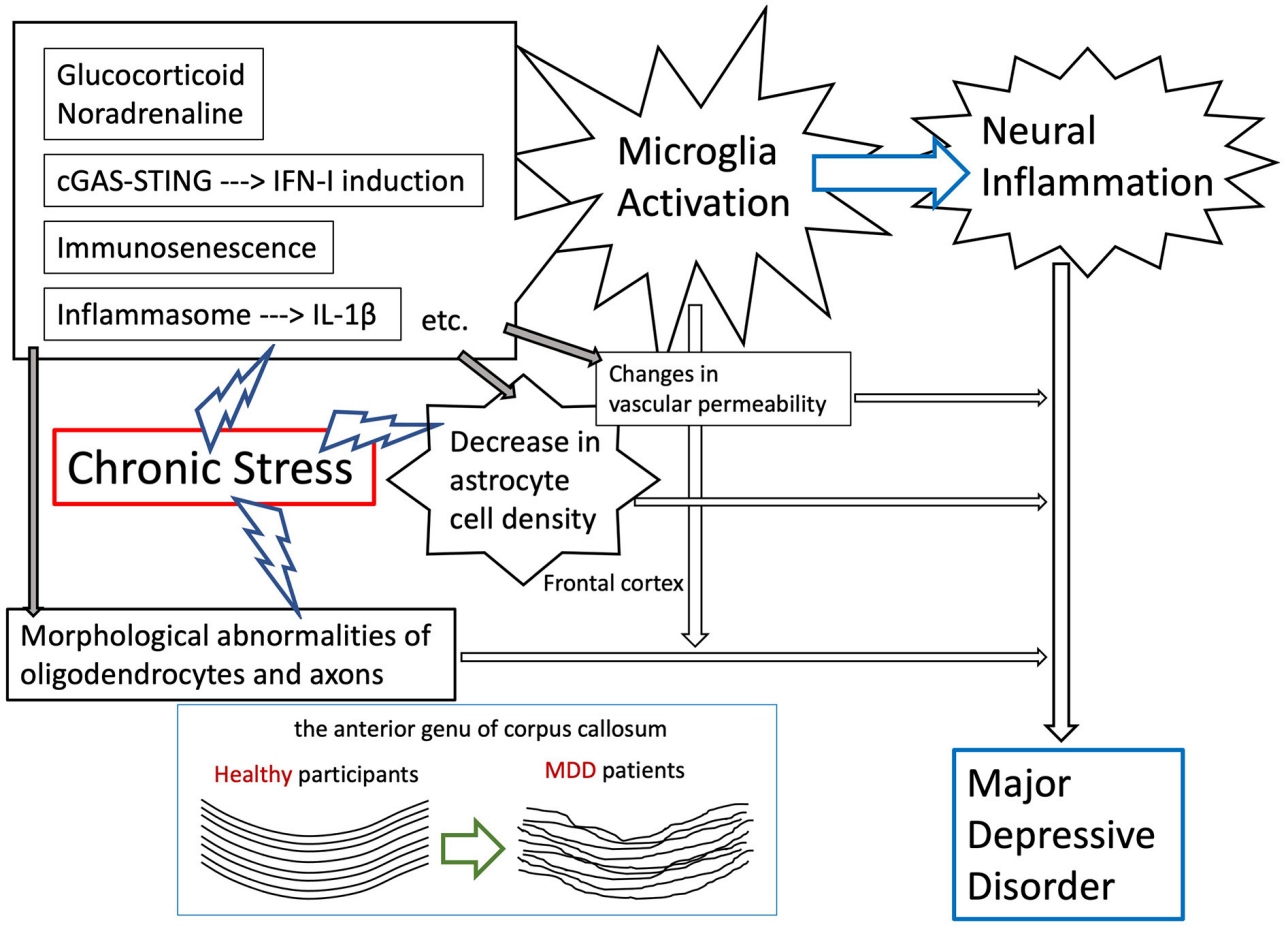
Expected mechanisms of the onset of MDD by the effects of intracerebral inflammation due to environmental stress exposure.

A primary goal of future studies is to understand the mechanisms of several brain/peripheral inflammations, which are discussed in this review, and the molecular mechanisms of neurons, oligodendrocytes/myelin, astrocytes, microglia cells, and vascular systems functional changes in chronic stress-exposed mouse models (Figure 1). Therefore, it will be intriguing to examine whether some factors involved in the inflammatory response, which were found in our studies, play roles in the compromised white matter/myelin integrity that has been reported.

## Author contributions

SM, YI, SS, and MT contributed to the search, assessment of available literature, and interpreted the results of previous studies. SM wrote the manuscript. All authors have approved the final version of the manuscript prior to submission.

## Funding

This study was supported in part by Grants from the Japan Society for the Grant-in-Aid for Scientific Research (C) 19K06916, 16K07073, AMED iD3 Grant Number DNW-14010, the AMED Translational Research Grant Number A-131, the Japan Foundation for Applied Enzymology, and the KINDAI COVID-19 Control Support Project.

## Conflict of interest

The authors declare that the research was conducted in the absence of any commercial or financial relationships that could be construed as a potential conflict of interest.

## Publisher's note

All claims expressed in this article are solely those of the authors and do not necessarily represent those of their affiliated organizations, or those of the publisher, the editors and the reviewers. Any product that may be evaluated in this article, or claim that may be made by its manufacturer, is not guaranteed or endorsed by the publisher.
